# Cognitive Training Improves Disconnected Limbs’ Mental Representation and Peripersonal Space after Spinal Cord Injury

**DOI:** 10.3390/ijerph18189589

**Published:** 2021-09-12

**Authors:** Valentina Moro, Michela Corbella, Silvio Ionta, Federico Ferrari, Michele Scandola

**Affiliations:** 1 NPSY-Lab.VR, Human Sciences Department, University of Verona, 37129 Verona, Italy; michela.corbella@univr.it; 2 Department of Rehabilitation, IRCCS Sacro Cuore “Don Calabria” Hospital, Negrar, 37024 Verona, Italy; federico.ferrari@sacrocuore.it; 3 Sensory-Motor Lab (SeMoLa), Department of Ophthalmology, University of Lausanne, Jules Gonin Eye Hospital-Fondation Asile des Aveugles, 1015 Lausanne, Switzerland; silvio.ionta@fa2.ch

**Keywords:** spinal cord injury, motor imagery, body representation, peripersonal space, rehabilitation

## Abstract

Paraplegia following spinal cord injury (SCI) affects the mental representation and peripersonal space of the paralysed body parts (i.e., lower limbs). Physical rehabilitation programs can improve these aspects, but the benefits are mostly partial and short-lasting. These limits could be due to the absence of trainings focused on SCI-induced cognitive deficits combined with traditional physical rehabilitation. To test this hypothesis, we assessed in 15 SCI-individuals the effects of adding cognitive recovery protocols (motor imagery–MI) to standard physical rehabilitation programs (Motor + MI training) on mental body representations and space representations, with respect to physical rehabilitation alone (control training). Each training comprised at least eight sessions administered over two weeks. The status of participants’ mental body representation and peripersonal space was assessed at three time points: before the training (T0), after the training (T1), and in a follow-up assessment one month later (T2). The Motor + MI training induced short-term recovery of peripersonal space that however did not persist at T2. Body representation showed a slower neuroplastic recovery at T2, without differences between Motor and the Motor + MI. These results show that body and space representations are plastic after lesions, and open new rehabilitation perspectives.

## 1. Introduction

Motor Imagery (MI) can be defined as the mental rehearsal of a movement without its physical execution [1]. It is associated with largely overlapping neural activation in the sensorimotor brain regions that would be used if the action was actually executed, in particular the pre-motor areas, the left intraparietal sulcus, the cerebellum and the basal ganglia [2,3,4,5,6]. However, in motor imagery, the activation of this network is reduced as compared to during the actual execution of a movement [7].

Evidence of the close connection between MI and motor execution can be found in the results of a number of studies on several motor-deprived conditions which indicate that MI is also impaired, for example in Locked-in syndrome [8], amyotrophic lateral sclerosis [9], dystonia [10] and chronic pain conditions [11,12].

Similar impairments have been also reported in Spinal Cord Injury (SCI). SCI causes a brain–body disconnection of the body parts below the spinal cord lesion with the interruption of the spinal efferences and afferences coming from and going to the brain. These lesions may be complete (i.e., with no lower-lesion sensory or motor functions spared) or incomplete (i.e., with some spared lower-lesion sensory-motor functions). The level of the lesion is indicated by the Neurological Level of Injury (NLI) that defines the portion of body impaired by the SCI. In patients with high cervical lesions, the face and head regions are normally connected to the brain while the body regions below the neck are disconnected from the brain (i.e., tetraplegia) and in patients with lesions affecting the lumbar region, the deprivation involves exclusively the lower parts of the body (paraplegia). The NLI indicates the most rostral spinal cord segment connected with the part of the body with preserved sensory-motor functions [13].

The impact of these sensorimotor deficits is devastating in terms of the sufferer’s ability to perform daily life activities (such as walking, working, or managing household activities, as well as sphincter control and sexual function), especially taking into consideration the fact that spinal cord injuries often involve people of working age (mean age: 54 [14]).

Furthermore, de-afferentation and de-efferentation cause indirect plasticity processes in brain networks that extend beyond the sensorimotor system (for reviews see: [15,16,17]), and probably also contribute to cognitive changes. Experimental studies have indeed demonstrated changes in SCI people’s representations of body [18,19,20,21], action [22,23,24] and space [20,25,26,27], which are topographically organised and as such reflect the topography of the disconnected body parts.

Deficits in motor imagery have also been found in cases of SCI [28,29] involving modifications in MI strategies [30], neuro-functional anomalies in the dynamics of event-related potentials [31], altered cortical activation [32,33,34] and altered functional connectivity [35]. Again, a link with the sensorimotor system is suggested by the topography of these modifications [29] which mainly affect motor imagery relating to actions involving the paralysed limbs. MI is thus closely associated with body representations and also with the representation of the body in the surrounding space, as suggested by the tendency of SCI people to imagine actions from an external, third person perspective rather than a first person perspective [29]. All this indicates that MI is also linked to body representation. In a recent study, Conson and colleagues [36] carried out a body representation task in which the participants were requested to judge the laterality of images showing their own or other people’s hands presented at various degrees of rotation. The use of MI strategies led to an advantage in terms of an improvement in the participants’ ability to recognise laterality in two conditions, one with their own hand, and one with another person’s hand. Similarly, the inhibition of multisensory integrative brain regions differentially affects the mental representation of own versus other people’s face images [37]. 

MI is also connected with peripersonal space representation, the representation of the space surrounding the body within which individuals can interact with the environment [38,39]. For example, during a MI task referring to an athletic gesture, expert fencers showed stronger cortical excitability of the proximal muscles (that are involved in handling a sword) than in the distal muscles. In addition, they manifested a stronger representation of their sword within their peripersonal space [40]. Crucially, cortical excitability during MI positively correlated with the representation of the sword within the individual’s peripersonal space [40].

A further step in this field of research regards the potential of using this body of knowledge in rehabilitation.

In recent decades, MI has been made use of in several rehabilitative training procedures [41,42] with mixed results [43]. MI training has been also used with SCI patients with some success, suggesting its efficacy in motor recovery, for example in the rehabilitation of reach-to-grasp movements [44], improving wrist extensions [45], and, despite mixed results, in neuropathic pain reduction [46].

On the other hand, recent evidence has shown that with SCI participants, motor intervention is efficacious in order to improve Body Representation [47] and Peripersonal Space [25,26].

In a study carried out by our group, the SCI participants performed a Mental Body Rotation task (a measure of Body representations [47]) before and after a standard motor rehabilitation program. In this task, the participants were requested to indicate whether images of rotated body parts showed a left or right body part. In healthy subjects, this task typically results in slower reaction times for body parts that are rotated 180°. This is due to the influence of the individual’s body representation since the participants internally simulated the rotation of the body in order to respond, and the greater the rotation of the image, the longer it took them to mentally simulate rotation. Before the intervention, the SCI participants had typically slower reaction times for rotated images of hands but not for images of feet. After training, even though the SCI individuals’ response times for the images of feet more closely resembled those of the healthy controls, they were still significantly slower [47]. These findings indicate that while physical training can produce benefits which possibly extend to cognitive levels (mental body representation), these benefits seem to remain partial (i.e., the response times are still slower) and are in any case confined to short-time periods.

In another study, the representation of the peripersonal space around the feet was found to have shrunk in the SCI participants when compared to the healthy controls [25]. However, passive mobilisation of their legs led to a recovery of this representation [25]. Surprisingly, even though the participants in the study presented with complete lesions and therefore could not feel the mobilisation of their lower limbs, actual passive motion was necessary for recovery [26].

To sum up, previous evidence suggests that despite the fact that physical training seems to improve body representation and peripersonal space in SCI individuals, these benefits might be too partial and/or short-lasting. These limitations may be due to the fact that traditional approaches comprise only physical training programs, and thus overlook the importance of mental/cognitive re-training to support any physical improvements. To test this hypothesis, the present study explored whether adding MI to standard physical training would improve the mental body representation of deafferented body parts and the peripersonal space around them.

The purpose of this study is to compare any improvements in mental body representation and peripersonal space in SCI patients undergoing a motor rehabilitation program training coupled or not with a mental imagery training.

## 2. Materials and Methods

### 2.1. Participants

People suffering from spinal cord injury were recruited on a voluntary basis thanks to the cooperation of the Spinal Unit of the IRCSS Sacro Cuore Hospital (Negrar, Verona, Italy) which is part of the International Group for Research into Spinal cord injury (https://sites.hss.univr.it/npsy-labvr/spinal-cord-injury-research-center/, accessed on 8 September 2021).

The inclusion criteria were (I) the presence of a traumatic spinal cord injury in a chronic phase (> 1 year); (II) the absence of sensory-motor impairments in the upper limbs; (III) age ≥ 18 years old; (IV) the absence of other neurological pathologies; (V) the absence of visual deficits and (VI) the age of the participant at the lesion onset ≥ 18 years old.

The exclusion criteria were (I) the presence of psychiatric or other neurological disorders and/or (II) spinal cord injury due to degenerative, neuroplastic or vascular causes.

The inclusion and exclusion criteria were applied by the authors FF and MC.

A total of 16 participants took part in the study (2 females, mean age = 51.22 SD = 9.88). All of them suffered from chronic spinal cord lesions (lesion onset mean = 21.22 years, SD = 9.81). The neurological level of the lesions ranged from C5 to L1, with 13 of the participants presenting with complete lesions (ASIA Impairment Scale = A) and three with incomplete lesions (ASIA Impairment Scale = C or D). One participant was excluded after the collection of the data due to a malfunctioning of the exoskeleton, that interrupted the participant’s rehabilitation cycle. The clinical and demographic data are detailed in Table 1.

All the participants read and signed the consent form. The project was approved by the Ethics Committee of the province of Verona (protocol n. 26902) and was conducted in accordance with the ethical standards of the 2013 Declaration of Helsinki.

### 2.2. Overall Design

**Figure 1:**
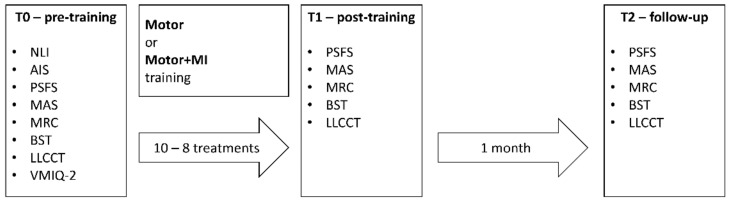
The 4 phases of the study. Evaluations carried out at T0, T1 and T2. NLI = Neurological Level of Injury [13]; AIS = ASIA Impairment Scale [48]; PSFS = Penn Spasms Frequency Scale [49]; MAS = Modified Ashworth Scale [50]; MRC = Medical Council Research scale [51]; VMIQ-2 = modified Vividness of Motor Imagery Questionnaire 2 [29]; BST = Body Sidedness Task [52,53]; LLCCT = Lower Limbs Crossmodal Congruency Task [25,26,54].

There were 4 steps: (I) pre-training evaluation (T0); a training phase which was either a motor-only treatment session (Motor), which acted as a control, or a “Motor Treatment and Motor Imagery” training session (Motor + MI); (II) post-training evaluation (T1) and (III) a follow-up evaluation one month after the final session (T2) (see Figure 1).

The scales and tasks used to assess the participants are described in the “Materials” section, and the objective was to investigate any changes both in each individual’s clinical symptoms, and in their body representation and their representation of the space surrounding their body. The descriptions of the two training sessions are reported in the “Procedures” section.

### 2.3. Materials

#### 2.3.1. Questionnaires and Clinical Scales

From all participants the following information were collected only in the pre-training session (T0):

1. The Neurological Level of Injury (NLI), that coincides with the most caudal part of the spinal cord with completely spared sensorimotor functions [13].
2. The ASIA Impairment Scale (AIS), that is a 5-point scale concerning the completeness of the lesion [48].
3. The Vividness of Motor Imagery Questionnaire-2 (VMIQ—Second Version) [55,56] is a measure of an individual’s capacity to imagine actions. In the present study, it was administered in the version adapted for spinal cord injured people [29] only at T0 with the aim of identifying potential correlations between the patient’s imagery capacity and any effects of the interventions carried out.
  It assesses three components of motor imagery: (I) visual imagery from a first person perspective (i.e., subjects are asked to visualise their body performing the action as if they were inside their body watching it with their own eyes; (II) visual imagery from a third person perspective (i.e., subjects are asked to visualise their body performing the action as if they were watching themselves from an external position such as in a mirror) or (III) Kinesthetic imagery, KIN (i.e., subjects are asked to simulate the musculo-skeletal sensations generated by executing the actions). These activate partially different processes [57,58,59], with KIN probably being the most sensitive measure of Motor Imagery.

The following questionnaires and clinical scales were collected at T0, T1 and T2, because we hypothesized that they might be potentially modulated by the training:

1. The Penn Spasms Frequency Scale (PSFS) [49] is used to estimate the intensity and frequency of spasms as reported by the patient.
2. The Ashworth Scale-Modified (MAS) [50] is used by clinicians to assess the presence and degree of spasticity on a 5 point scale.
3. The Medical Research Council (MRC) scale [51] is used to assess the muscular strength of the right and left legs in movements involving: the flexion, extension and abduction of the hips; the extension of the knee and the dorsal and plantar flexion of the ankle.

#### 2.3.2. Lower Limbs Crossmodal Congruency Task (LLCCT)

The LLCCT is an experimental task which is used to estimate the representation of peripersonal space (PPS) around the lower limbs [60,61,62]. It was used in the present study at T0, T1 and T2 to ascertain whether there was any modulation in the participants’ representation of peripersonal space as a result of the interventions.

In this task, tactile stimulations were administered by means of two stimulator devices which participants held in their hands (DAEX25 Sound Exciter Pairs, Dayton Audio, Pleasant Valley, OH, USA). These are small cylinders which can be made to vibrate independently of each other at four contact points on the device: top left-hand side, top right-hand side, bottom left hand side and bottom right hand side. The participants held the two tactile stimulators (one in each hand) between their index fingers and thumbs, with their index finger on the top and their thumb on the bottom of the device.

**Figure 2:**
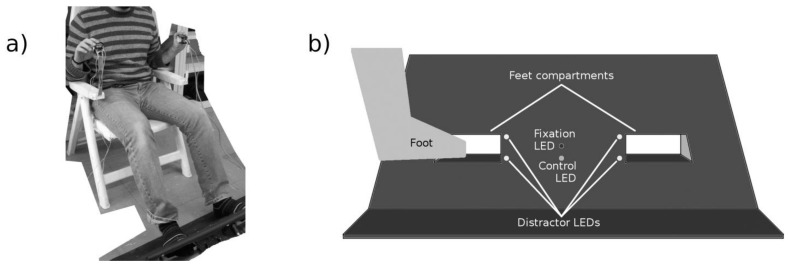
The Lower Limbs Crossmodal Congruency Task. (**a**) Position of the participants during the CCE evaluation; (**b**) Schematic representation of the frontal part of the wooden frame used for the visual stimuli (LEDs) on the inside edge of the foot compartments. The representation of the foot in the image is not in an anatomical position. During the experiment the participants were in front of the wooden frame and the feet were inserted into the compartments.

The participants’ feet were inserted into a pre-tested custom-made apparatus [25,26] (Figure 2a). The apparatus consisted of a wooden frame (80 × 38 cm, tilted at 30° from the vertical plane) with two compartments for the feet (each 15 × 8 cm, distance 22 cm, Figure 2b) and four LEDs (two for each foot compartment) positioned at the lower and upper inner corners of the foot compartments. These LEDs gave visual stimuli that function as distractors (with respect to the tactile stimulation). Two further LEDs (one red and one green) were placed in the centre of the wooden frame between the foot compartments, with the red light functioning as a fixation point and the green light as a control to check for the participant’s attention. In some additional catch trials, the green light flashed and participants were requested to report this. In this way we made sure that they were actually looking at the lights.

**Figure 3:**
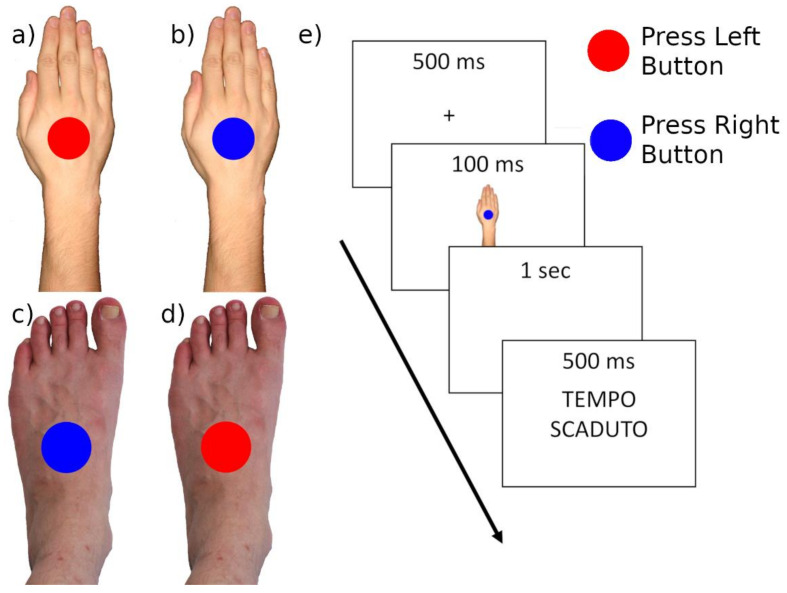
Body Sidedness Task. (**a**) Incongruent hand stimulus; (**b**) Congruent hand Stimulus; (**c**) Incongruent foot stimulus; (**d**) Congruent foot stimulus; (**e**) Experimental trial timeline. The last slide “Time up” (in Italian—tempo scaduto) is shown only if the participant did not answer within 1 s.

Tactile stimuli (three 50 ms vibrations separated by 50 ms gaps, total duration 250 ms) were administered by means of the handheld stimulation devices (i.e., either near the index finger or the thumb) and on either the left hand or right hand side of the device (i.e., stimulating either the left or the right hand). 30 ms before each tactile stimulus, a visual distractor stimuli was displayed by an LED on the foot compartments, either the one on the upper-left, or on the upper-right, or on the lower-left, or on the lower-right corner (upper: close to the dorsal part of the foot, lower: close to the plantar part of the foot, see Figure 3b) [63].

Visuo-tactile trials were classified as Congruent or Incongruent, depending on whether the tactile and visual stimulations were both at the top or both at the bottom positions (Congruent) of the handheld device and the foot compartment respectively, or in different positions (i.e., one on the top and one on the bottom, Incongruent) and whether the visual distraction stimulus and the tactile target stimulus were Homolateral (both on the same side of the body) or Bilateral (on different sides of the body). The participants were requested to focus on the red fixation LED on the wooden frame and verbally report (as quickly as possible) where they felt the tactile stimulus on their hand (i.e., “upper” or “lower” position; responses: “TAH” or “TOH”, respectively), irrespective of which hand the stimulus was administered to. They were also instructed to ignore the visual distractors.

The difference in reaction times between that when the tactile stimuli are congruent with the stimuli near the feet (i.e., both the hand and feet stimuli in the upper position or both in the lower position) and that when the positions of the hand and feet stimuli are different (incongruent) is known as the Congruency Effect. This effect is greater when the visual stimuli are within the participant’s PPS (i.e., the same side of the body with respect to the tactile stimulus, homolateral) and smaller when they are outside the PPS (on opposite sides of the body, i.e., bilateral) [62,64,65]. In fact, the two visual and tactile stimuli presented on the same side of the body are processed as if they were inside the PPS around that side of the body, while when they are bilateral, that is, on opposite sides of the body (e.g., a tactile stimulus on the left hand and a visual stimulus on the right foot), the visual stimulus is processed as if they were outside the PPS of the body part stimulated by the tactile stimulator [25,26,54,60,66]. The Congruency Effect was thus greater in the homolateral trials than in the bilateral trials (measured in terms of the differences in response times) and is considered an effect of the presence of PPS representation.

During the task, the wooden frame was placed on the floor in front of participants, with the distance adjusted according to the length of each participant’s legs (never less than 30 cm). There were two stimulation conditions: in the REAL condition participants were helped to place their feet into the two compartments, while in the VOID condition the feet were placed outside the compartments and covered in order to be out of sight. The VOID condition was necessary as a control condition to verify any potential effects of the stimulation on the representation of space far from the body.

In order to ensure that the participants focused on the centrally positioned LEDs, they were instructed to say the word “luci” (the Italian word for “lights”) whenever they saw the green LED flashing (control trials), while to guarantee that they paid attention to the tactile stimuli, false stimulations were administered. These false stimulations consisted of a distractor light on the wooden frame which flashed without tactile stimulation. In these cases, the response was expected to be “niente” (the Italian word for “nothing”). The PPS task consisted of 162 trials for each block, for a total of about 15 min.

#### 2.3.3. Body Sidedness Task (BST)

This experimental task was used to estimate any potential changes in the participant’s body representation induced by the interventions. It was carried out at T0 and repeated at T1 and T2.

The BST [52,53] is a Simon-like task [67] in which a blue or red target circle is presented at the centre of a screen. The circle is superimposed on an image showing a left or right part of the body. The participant is asked to press, as fast as possible, a button with his/her left hand when the circle is red, and another button with their right hand when it is blue, irrespective of the side of the body part seen in the image. In this way, in each trial, the body part and the circle colour can be congruent (i.e., a left hand or foot when the circle is red, a right hand or foot when the circle is blue) or incongruent (i.e., a right hand or foot when the circle is red, a left hand or foot when the circle is blue).

An incongruent body part shown in the background leads participants to be more prone to errors and slows down their reaction times [52,53]. This effect is caused by the presence of an internal body representation that, if it is preserved, conflicts with the incongruent condition.

In the version of the BST used in this study, the participants were requested to respond (as fast as possible) to the red circles pressing the keyboards “Q” with their left hand and to the blue circles pressing the “P” with the right hand. There were two blocks of tests, characterised by different backgrounds: a dorsum picture of a Hand or a Foot. The blocks were counterbalanced across participants, using the “Foot—Hand—Hand—Foot” or “Hand—Foot—Foot—Hand” block sequences. See Figure 3a–d for some examples of the stimuli and Figure 3e for the timeline of a single trial.

Each trial started with a 500 ms fixation cross, followed by the stimulus that was shown for 100 ms. Participants were asked to give their response within 1 s. If they did not answer within this interval of time, a written feedback “Time up” appeared and lasted for 500 ms. (Figure 3e).

The whole experiment was composed of 96 trials, for a total duration of about 10 min. This experiment was written in Visual C# (Microsoft) which recorded accuracies and reaction times for each trial.

### 2.4. Procedure

The participants were randomly assigned to either the “Motor” or the “Motor + MI” groups.

Irrespective of the group to which they had been assigned, all of them participated in a 2 week intervention involving motor rehabilitation (with exoskeleton or passive mobilisation) for a total of 10 sessions (in the case of technical constraints, a minimum of at least 8 sessions was guaranteed). The decision whether to participate in the exoskeleton-based or the passive mobilisation-based motor training sessions was taken by the participants themselves.

Those who had chosen exoskeleton training did assisted walking with an EKSO (Ekso GT^TM^ (https://eksobionics.com/, accessed on 8 September 2021)) which was totally automated (i.e., no active muscular activity was required). This was carried out in the rehabilitation rooms of the hospital. The movements were completely passive even though they mimicked a real sequence of steps.

**Figure 4:**
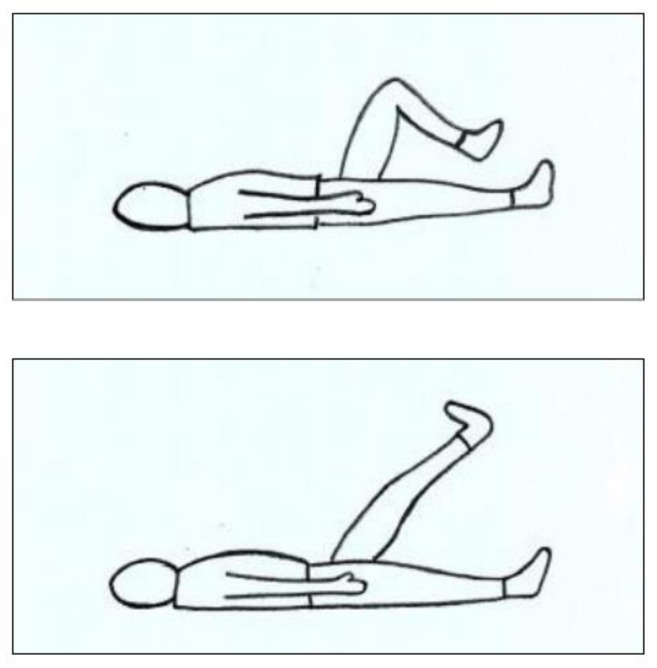
Schematic representation of passive mobilisation.

The motor training with passive mobilisation consisted of flexion-extension movements of the lower limbs at all the joint levels (hip, knee, and ankle) and for the entire range of motion (see Figure 4). These movements were passively induced by a physiotherapist and simulated the Exoskeleton induced movement, with the difference that the participant was not standing but lying on a rehabilitation bed.

Only the “Motor + MI” group did motor imagery training in addition to one of the motor intervention activities described above.

The training comprised a task in which the participants were requested to keep their eyes closed and to imagine themselves while they were executing the action that was denominated by the examiner. They were asked to press a key both when the imagined action started and when it ended. There were 12 different actions, 4 involving upper limbs, 4 involving lower limbs and 4 involving the whole body. The task was repeated twice, for a total of 24 trials. In the same week, there were 5 different types of actions in order to prevent learning effects and to maintain the participant’s attention.

Each single Motor Treatment session (either exoskeleton or passive mobilisation) lasted 30 min, while the motor imagery training sessions lasted 15 min. In the “Motor + MI” group, the two interventions were administered in succession.

### 2.5. Data Handling and Statistical Analysis 

All the statistical analyses were conducted within the Bayesian Framework [68,69,70,71,72].

For statistical inference, the Savage-Dickey Bayes Factors (BF_10_) were computed [73,74] by using the package logspline 2.1.15 [75]. Traditionally, with a BF_10_ > 3 the alternative hypothesis is considered valid, while the null hypothesis is considered valid when there is a BF_10_ < 1/3 [76]. However, taking into account our small sample size, we decided to use as thresholds BF_10_ > 5 for the alternative hypothesis, and < 1/5 for the null one, as suggested in [77].

For all of the Bayesian Models, a Posterior Predictive *p*-value (ppp) [78] and the Effective Number of Simulation draws (n_eff_) [69] (pp. 286–287) are provided, with the former being a qualitative score that should be around 0.5 if the model represents the data (ppp ≈ 0.5) [78], and the latter being the total number of stationary MCMC iterations, corrected by the autocorrelation among the four MCMCs (n_eff_ > 10) [69] (pp. 286–287).

The prior distributions for the effects of the models are normal distributions with a variance of 5, since this is a non-standardised effect size that might highlight a clear effect in both ordinal and continuous variables. With ordinal variables it means that there is a difference of—at least—the 45% in the evaluations, while with continuous variables this means that the difference has a standard deviation of about 3 [79].

After the main analyses, further testing, when needed (namely where there were effects wih BF_10_ > 5 that describe the behaviour on more than two levels), were computed on the marginal posterior distributions. These marginal posterior distributions come from the summation and subtraction (according to the contrast matrix of the population-level effects) of the posterior distributions of the fixed-effects. For this reason, the marginal posterior distributions were tested by means of a-posteriori distribution percentages [80].

To keep the same level of confidence used with Bayes Factors, the percentages that are considered as an index of an effect are ≥ 83.5% and ≤ 16.5% (since 83.5/16.5 ≅ 5). Descriptive statistics on Bayesian analyses are presented in terms of mode and the 95% Highest Density Interval (HDI) [72] (pp. 87–89) of the marginal posterior distribution with this format: mode {HDI lower boundary, HDI upper boundary}.

Further details in Appendix A.

#### 2.5.1. VMIQ-2, PSFS and Clinical Data Analysis

The scores at the VMIQ-2, namely the motor imagery scores in the kinesthetic (KIN), first-person (1PP) and third-person (3PP) perspectives, were compared between the two groups, using two factors: Group (Motor, Motor + MI) and Perspective (1PP, 3PP and kinesthetic). The PSFS (frequency and intensity), MRC and MAS scores were analysed using two factors: Group and Time (T0, T1, T2). For Lesion Onset and Age, only the factor Group was taken into consideration.

We used either a Bayesian Ordinal Model (VMIQ-2, NLI, PSFS frequency and intensity scores, MAS and MRC) or a Bayesian Robust Linear Model (Age, Lesion onset), according to the ordinal or continuous nature of the data. The JAGS code of the models is shown in Appendix B.

#### 2.5.2. LLCCT Analyses

The Congruency Effects (namely, the differences between the Incongruent and Congruent trials) resulting from the data of this experimental task were used to test the effects of the rehabilitation training sessions. The REAL condition was analysed separately from the VOID condition in order to simplify the presentation of the results. Note that the REAL condition shows the effects that are connected with lower limb PPS representation, while the VOID condition is a control condition.

In order to use the whole data variance, to respect the ex-Gaussian nature of the reaction times [81], and to adequately consider population-level effects (also known as fixed effects) and group-level effects (also known as random or varying effects), a tailored, specific Generalised Multilevel Linear Model was used. This model [26] (see Appendix C for the JAGS code) estimates the effects of the population-level effects on the Congruency Effect from the simple reaction times.

In both of the analyses (REAL and VOID), the population-level effects were: Space (homolateral, bilateral), Time (T0, T1, T2) as linear and quadratic components (to capture non-linear effects), and Group (Motor, Motor + MI). The group-level effects were: Time as linear and quadratic components, and Space grouped by participant

Moreover, in order to verify the potential role of clinical variables, further analyses were carried out but only on data from the trials of the Real condition with homolateral stimuli at T1, considering as covariates the NLI, lesion onset, the Intensity and Frequency of PSFS and the score on the VMIQ2 scale. We chose the homolateral trials in the Real condition as these represent the PPS representation effect.

All covariates were converted into z-scores to avoid potential biases.

The JAGS code of the model is shown in Appendix D.

#### 2.5.3. BST Analyses

As described in the methods section, the Congruency Effects were of interest in order to test the effects of rehabilitation on body representation. The same models used for the LLCCT analysis were carried out here.

The population-level effects were: Time (T0, T1, T2) as linear and quadratic components, to capture non-linear effects, Background (Hand, Foot) and Group (Motor, Motor + MI) and all their interactions. The group-level effects were Background and the linear and quadratic components of Time, grouped by participant.

Moreover, further analyses were only executed on trials with the Foot background (where a modulation was expected) at T1, considering as covariates the z-scores of NLI, lesion onset, the Intensity and Frequency of PSFS, and the score on the VMIQ2 scale.

## 3. Results

All of the models showed ppp scores between 0.58 and 0.39, suggesting that the models represented the data; furthermore, all the effects had Ȓ < 1.1 and n_eff_ > 10, indicating the reliability of the posterior distributions.

### 3.1. General Imagery Ability—VMIQ-2, PSFS and Clinical Data Results

In the T0 assessment, no differences were observed for NLI (BF_10_ = 0.15), Lesion Onset (BF_10_ = 1.01) and Age (BF_10_ = 0.22) between the two groups, meaning that they were thus comparable. In the VMIQ-2 analysis, all of the effects showed the validity of the null hypothesis (all BF_10_ < 0.15), indicating the absence of differences for the three perspectives (1PP, 3PP, Kinesthesic). For this reason, in subsequent analyses in which the VMIQ-2 scores were used as covariates, an average score for each participant for the three perspectives was considered.

The clinical variables did not vary over time.

The MRC scores did not indicate any effects of Time (linear BF_10_ = 0.04 and quadratic BF_10_ = 0.03), nor were there any effects relating to the interaction between Time and Group (linear BF_10_ = 0.03 and quadratic BF_10_ = 0.04). However, a small difference between the “Motor + MI” (1 {1, 1} MRC scale) and “Motor” (1 {1, 3} MRC scale) was observable (BF_10_ = 134.92).

In both the Intensity and Frequency scores of the PSFS, none of the effects reached the alternative hypothesis boundary (all BF_10_ ≤ 0.34). For this reason, only PSFS scores at T0 were used as covariates in the subsequent analyses.

The Modified Ashworth Scale scores (assessing spasticity) did not show any changes in Time (linear BF_10_ = 0.03 and quadratic BF_10_ = 0.04), Group (BF_10_ = 0.30) or the interaction between these (linear BF_10_ = 0.04 and quadratic BF_10_ = 0.03).

### 3.2. LLCCT Results

In both the REAL and VOID conditions (Table 2), the Space:Group:Time^2^ effects reached BF_10_ > 5 suggesting the validity of the alternative hypothesis in both cases, namely the differences between Homolateral and Bilateral trials vary between groups and over time.

When we tested for differences between the Homolateral and Bilateral trials (corresponding to PPS and extrapersonal space), we found that in the REAL condition the PPS representation (Homolateral > Bilateral) is only present in the “Motor + MI” group and only at T1 (Pr(x > 0) = 99.99%, Homolateral = 39.07 {27.54, 48.92}, Bilateral = 10.15 {−1.41, 21.57}) This suggests a temporary recovery of the Lower Limb PPS representation due to the intervention. In contrast, in T0 and T2, the responses in the Bilateral trials (T0 = 1.78 {−7.48, 12.99}, T2 = 34.00 {23.15, 44.36}) were slower than the Homolateral trials (Pr(x > 0) = 13.46% and Pr(x > 0) = 1.14%, respectively. T0 = −6.17 {−15.18, 3.23}, T2 = 17.15 {7.78, 27.80}).

**Figure 5:**
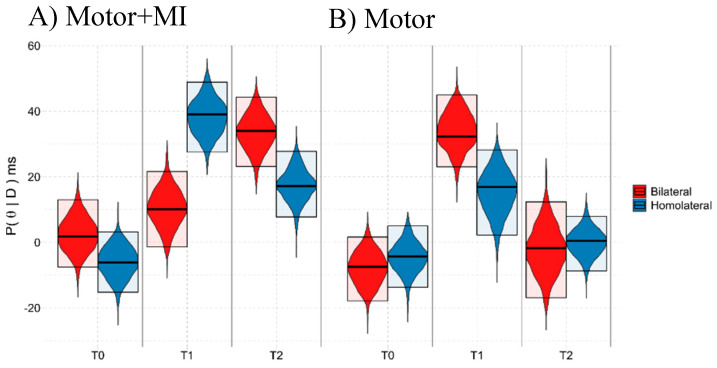
Graphical representation of the marginal posterior distributions of the Space:Condition:Time interaction in the REAL condition. The violin plots represent the marginal posterior distribution of the Bayesian model, the upper and lower boundaries of the box show the limits of the 95% HDI, while the central line represents the distribution mode. On the y−axis, the marginal posterior distribution (P(θ|D)), in milliseconds, represents the performance of the participants, and the x−axis shows the Time points: T0 (pre training), T1 (post training) and T2 (follow-up). (**A**) = Motor + MI, (**B**) = Motor. H1 = Pr(x > 0) > 83.5%, meaning that Homolateral > Bilateral, index of PPS representation. H2 = Pr(x > 0) < 16.5%, meaning that Bilateral > Homolateral, and with no PPS representation.

In the case of the “Motor” group, the presence of a PPS representation relating to the Lower Limbs did not emerge either before or after the training sessions (Pr(x > 0) T0 = 65.57%, Bilateral T0 = −7.41 {−17.86, 1.63}, Homolateral T0 = −4.32 {−13.70, 5.01}; Pr(x > 0) T2 = 61.52% Bilateral = −1.76 {−16.87, 12.36}, Homolateral = 0.47 {−8.71, 7.97}). In T1, the reaction times were slower for the Bilateral than for the Homolateral trials (Pr(x > 0) = 1.79% Bilateral = 32.29 {23.01, 44.96}, Homolateral = 16.90 {2.23, 28.24}). For a graphical representation, see Figure 5.

In the VOID condition there was no recovery of Lower Limb PPS representation in T1 and T2. A description of the results is in Appendix E.

#### 3.2.1. Covariation with NLI, Lesion Onset, PSFS and VMIQ-2

The analysis showed that there were effects in the interactions between Group:PSFS—Frequency, Group:PSFS—Intensity and Group:NLI (see Table 3 for the effects).

**Figure 6:**
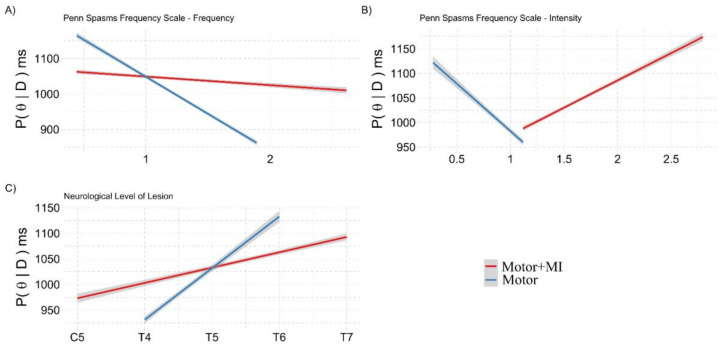
Graphical representation of the marginal posterior distributions of the interactions in the Covariation of the LLCCT paradigm in the REAL condition at T1 with NLI, PSFS and VMIQ-2 model. The y axis shows the marginal posterior distribution (P(θ|D)) in milliseconds, representing the performance of the participants. The *x*-axis refers to the covariates rescaled from z-scores to the original scores. All the graphical representations covariate the performance at the LLCCT task at T1 post-training (greater values on the *y*-axis represent a better PPS representation) with a different scale. The grey shading represents the 95% CI of the marginal posterior distributions. The *x*-axis of panels (**A**,**B**) shows the scores on the respective scales. The *x*-axis of the panel (**C**) shows the Neurological Level of Injury.

The frequency of spasms in the “Motor + MI” group shows that the PPS representation was more stable, while the frequency of spasms in the “Motor” group shows a detrimental effect (Figure 6A). The intensity of spasms impaired the PPS representation in the “Motor” group, while in the “Motor + MI” group there was a paradoxical improvement effect (Figure 6B).

The level of lesion (most rostral NLI) negatively affected the PPS representation in the “Motor” group, while in the “Motor + MI” group, the PPS representation was more stable (Figure 6C).

#### 3.2.2. Ad-Interim Discussion

Taken as a whole, these results indicate that the “Motor + MI” intervention is sufficient to increase the patients’ representation of the PPS around their lower limbs. Unfortunately, this recovery did not last until the follow-up assessment (T2). An analysis of the covariates showed that in the “Motor + MI” group, the PPS representation was more stable than in the “Motor” group in terms of the frequency of spasms and the neurological level of lesion (Figure 6A,C), while the PPS representation improves with greater spasms intensity.

### 3.3. BST Results

The posterior distributions of the effects of the two interactions show that there were effects on the Background:Time^2^, the Group:Time^2^ and the Background: Group interactions (see Table 4).

#### 3.3.1. Background:Time^2^

In order to understand whether the body part shown in the background modulated the participants’ responses, the marginal posterior distributions were first tested against zero. If the body part shown had an impact on the task, 83.5% of the distribution would be greater than zero.

While this happened for the trials with the Hand background in T0 and T1, and in T2 the performance was near the boundary (T0: Pr(x > 0) = 97.48%, 4.33 {0.10, 8.50}; T1: Pr(x > 0) = 92.60%, 3.01 {−1.14, 7.62}; T2: Pr(x > 0) = 80.16%, 1.67 {−2.29, 6.53}), in the trials with the Foot background, the performance was > 0 only in T2 (T2: Pr(x > 0) = 100%, 10.03 {5.04, 13.88}; T1: Pr(x > 0) = 30.04%, −1.37 {−6.01, 3.58}; T0: Pr(x > 0) = 39.28%, −0.070 {−5.00, 3.84}). This indicates that a recovery of the representation of the PPS relating to the feet only emerged some time after the intervention.

These results were confirmed by a comparison between the participants’ performances at the different timepoints with different backgrounds. In fact, while in the trials with the Hand background there was never a difference (T0–T1: Pr(x > 0) = 62.20%, 0.76 {−4.57, 7.64}; T0–T2: Pr(x > 0) = 74.94%, 2.68 {−3.86, 8.50}; T1–T2: Pr(x > 0) = 65.89%, 1.44 {−4.81, 7.52}), in the trials with the Foot background, the differences between T0–T2 and T1–T2 indicated that there was a better foot PPS representation at T2 (Pr(x > 0) = 0.08%, −9.69 {−16.54, −3.47}; Pr(x > 0) = 0.01%, −10.67 {−16.91, −4.57}, respectively), while the difference between T0 and T1 was not relevant (Pr(x > 0) = 57.86%, 0.27 {−5.68, 7.47}).

**Figure 7:**
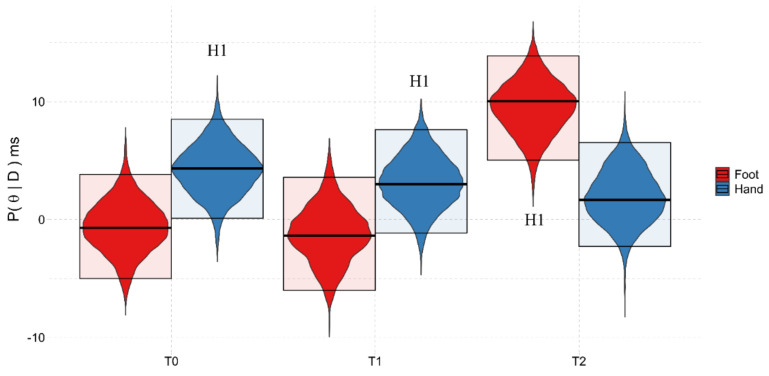
Graphical representation of the marginal posterior distributions (P(θ|D)) of the Background:Time^2^ interaction. Description as in Figure 4. H1 = Pr(x > 0) > 83.5%, meaning that the BSE is greater than zero, showing a preserved body representation. T0 = baseline evaluation; T1 = post-training evaluation; T2 = follow-up evaluation.

There was no evidence of any effect caused by the Group for this interaction (see Figure 7).

#### 3.3.2. Background:Group

**Figure 8:**
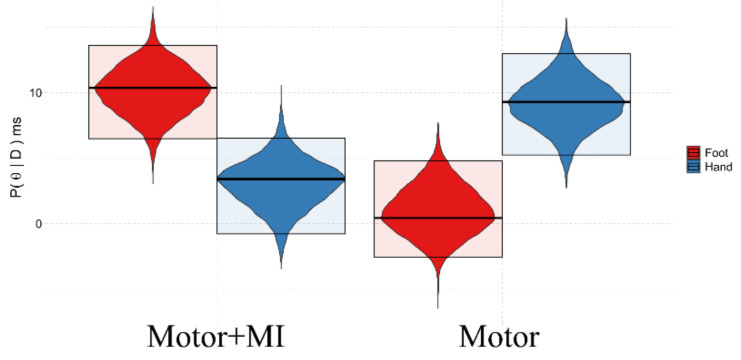
Graphical representation of the marginal posterior distributions (P(θ|D)) of the Background:Group interaction. Description as in Figure 4. H1 = Pr(x > 0) > 83.5%, meaning that the BSE is greater than zero, indicating a preserved body representation. Motor + MI = Motor Treatment and Motor Imagery group. Motor = Motor Treatment only group.

There was evidence of a difference between the two interventions in the background:group interaction (see Figure 8). In fact, taking into consideration the average measures relating to the three Time assessments, the results confirm that there is a PPS representation of the Hand in both the groups, (Motor + MI: Pr(x > 0) = 94.76%, 3.41 {−0.77, 6.52}; Motor: Pr(x > 0) = 100%, 9.29 {5.25, 12.96}), while the representation relating to the Feet is only present in the case of the “Motor +MII” group (Pr(x > 0) = 100%, 10.38 {6.50, 13.62}). In the “Motor” group (Pr(x > 0) = 68.51%, 8.52 {3.93, 14.24}), the results do not indicate a clear presence of body representation for the feet.

#### 3.3.3. Group:Time^2^

**Figure 9:**
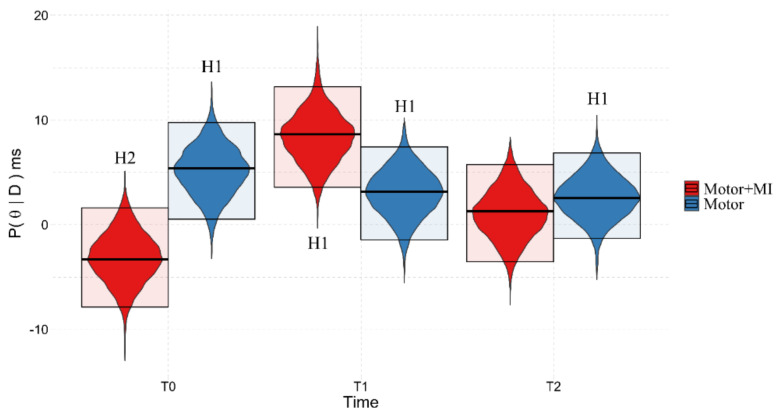
Graphical representation of the marginal posterior distributions (P(θ|D)) of the Group:Time interaction. Description as in Figure 4. H1 = Pr(x > 0) > 83.5%, meaning that the BSE is greater than zero, indicating a preserved body representation. H2 = Pr(x > 0) < 16.5%. T0 = baseline evaluation; T1 = post-training evaluation; T2 = follow-up evaluation. Motor + MI = Motor Treatment and Motor Imagery group. Motor = Motor Treatment only group.

This interaction did not involve the background displayed in the stimuli (see Figure 9). However, it is possible to observe that the participants in the “Motor + MI” group showed a general improvement in their body part representation at T1but it seemed to be a short-term effect and did not persist up to T2 (T0: Pr(x > 0) = 9.72%, −3.30 {−7.86, 1.61}; T1: Pr(x > 0) = 100%, 8.68 {3.59, 13.17}; T2: Pr(x > 0) = 66.74%, 1.29 {−3.55, 5.75}). In contrast, the performances of the “Motor” group were stable, with a global body representation always present (T0: Pr(x > 0) = 98.39%, 5.40 {0.52, 9.77}; T1: Pr(x > 0) = 90.88%, 3.15 {−1.44, 7.44}; T2: Pr(x > 0) = 90.16%, 2.55 {−1.32, 6.86}).

#### 3.3.4. Covariations with NLI, Lesion Onset, PSFS and VMIQ-2

**Figure 10:**
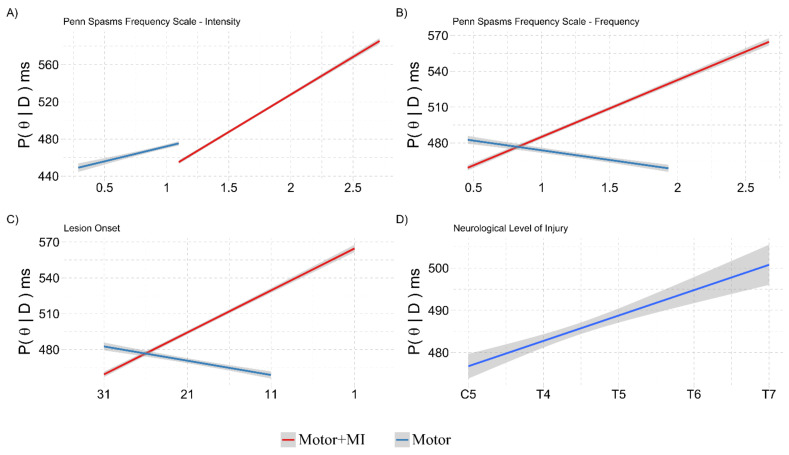
Graphical representation of the marginal posterior distributions (P(θ|D)) relating to the main effects and interactions in the Covariation of the BSE paradigm with Foot background at T1, with NLI, Lesion Onset PSFS and VMIQ-2 model. Slower values on the *y*-axis means a better body representation. The description is as in Figure 7. In panels (**A**,**B**) the *x*-axis shows the scores of the respective scales. In panel **C** the *x*-axis shows the number of years since the Lesion Onset and the beginning of the training, while in panel **D** the Neurological Level of injury. Motor + MI = Motor Treatment and Motor Imagery group. Motor = Motor Treatment only group.

These results are shown in Table 5 and suggest that the Interval from lesion Onset has an effect on the “Motor + MI” group with a loss of lower limbs representation over time since lesion onset, and more recent lesions having a better lower limbs representation than the older lesions (Figure 10C). Viceversa, in the “Motor” group interval from lesion onset does not change the body representation. 

In the ”Motor + MI” group there is again the effect seen in the PPS representation, where the higher frequencies and intensities of spasms lead to a better body representation of the lower limbs (Figure 10A,B). Finally, the NLI impacts the body representation, showing that lower lesions (and therefore a greater portion of body connected to the brain) lead to a better body representation of the lower limbs (Figure 10D).

#### 3.3.5. Ad-Interim Discussion

Taken as a whole, these results indicate post-training improvements in the body representation of the feet (delayed over time), while the representation of the hands remains constant (Background:Time^2^ interaction). This improvement was evident in the follow-up assessment, suggesting slow neuroplastic processes. Moreover, the recovery of the feet representation was stronger in the “Motor + MI” group (Group:Background interaction) than in the “Motor” group.

This latter group, however, showed indications of a better general body representation (Group:Time interaction) than the “Motor + MI” group.

Finally, we observed that at T1, there were better post-training body representations relating to the feet in cases with more recent lesions and more frequent and intense spasms in the “Motor + MI” group, and more caudal lesions in both groups.

## 4. Discussion

Two complementary results emerged from the present study. The first regards a confirmation that sensorimotor de-afferentation and de-efferentation extend their effects beyond the sensorimotor system towards cognitive functions and impact body and space representations [20,24,26]. The second, on the other hand, relates to the evidence that was found indicating that rehabilitation is able to reduce these deficits, in particular when associated with training involving the mental imagery of actions. Body and PPS representations seem to be at least partially independent, as a specific improvement in the representation of the PPS around SCI individuals’ limbs was recorded immediately after the intervention, but this did not last up to the follow-up assessment. In contrast, changes in the body representation relating to the lower limbs only became evident at the follow-up assessment. Finally, the improvements made did not depend on clinical variables, as no changes were recorded in muscular strength (as seen with the MRC scale) or spasticity (MAS scale). The representations were modulated by the intensity and frequency of spasms. Interestingly, the group who participated in the “Motor + MI” training was less affected by these covariates, but more sensitive to their positive influence.

To sum up, in line with the evidence available in the literature on the subject [25,26,47,82] indicating that lower body and PPS are topographically organised, the present study extends the knowledge provided in previous work by showing the importance of including cognitive training in rehabilitation programs in order to achieve better restoration of sensorimotor functions after SCI.

### 4.1. The Effects of Training on PPS

The representation of PPS has a high degree of plasticity and is strictly connected with action representation [83,84]. The processes associated with this plasticity are rapid in their extension but also in their shrinkage, and thus without specific training, the PPS representation around paralysed body parts may be lost.

Indeed, it is known that the representation of PPS can be easily extended by short-term actions and the use of tools, for example toy golf-clubs [61], a cane for blind individuals [85] or a rubber hand in experimental paradigms [60,86]. Moreover, PPS can also be extended when the object is positioned in discontinuity with respect to the body, as shown by the use of a computer mouse [87] and in virtual reality paradigms [88].

In terms of the aims of this study, it is noteworthy that there is evidence concerning the possibility that PPS can also shrink [89,90]. In particular, healthy participants whose upper limb is blocked for a whole day, show a PPS reduction [89,90], similar to the shrinkage in PPS representation found in the SCI participants in the present study [25]. Similar behaviour has been also reported in amputee individuals whose PPS representation includes the prosthesis when worn, but which shrinks to the stump when they are not wearing the prosthesis [91].

In contrast to previous studies [25,26], the motor treatment administered in this program is not sufficient in order for a PPS recovery to be achieved–mental imagery training is also required. The difference with respect to previous results [25,26] might depend on the variation in the time when the assessments were carried out. In fact, while in previous studies the estimation of PPS was done immediately after passive mobilisation, in this rehabilitation project, the evaluations were generally executed on a different day after the end of the training. This delay might have had a negative effect on the short-term PPS recovery when only motor training was administered, but not when this was integrated with motor imagery.

Altogether these results show the importance of PPS representation as a space for action and an interface between the individual and the environment around him or her [38,39]. There is, therefore, a possibility that a reduction in the PPS representation around the lower limbs in SCI individuals does not indicate the lack of a specific representation, but rather hides the existence of an extremely plastic cognitive representation, ready to emerge when action possibilities are cognitively conceived.

These effects merit further investigation, particularly taking into account the risks of falls and injuries during changes in position and posture that SCI individuals constantly face.

### 4.2. The Effects of Training on Body Representations

The BST is based on the automatic processes relating to the sidedness of body parts [52,53], in particular using images showing adjacent body parts (e.g., the forearm for a hand and the ankle for a foot) [52,53]. This specificity in the stimuli suggests that this task measures the topographical organisation of body representation, with particular reference to the local relations between body parts in a perceptual and body-centred perspective. This is referred to by some authors as the Body Structural Description [92,93,94] and by others as a component of Body Image [95,96].

Regardless of the definitions, the visuo-spatial and topographical representation of body parts contains information about local relationships between body parts despite continuous joint movements and transformations in the orientation of the body and/or body parts.

Our data suggest that without rehabilitative interventions, this representation is somato-topically impaired in SCI individuals. This is in contrast with a previous study [97] that did not find any distortion in a group of SCI participants. However, it is worth noting that the two tasks used in the studies are different. In fact, in [97] the SCI participants were asked to “draw” a whole body starting from a cue that showed various body parts. They were requested to identify the position of the body parts in the cues by anchoring them to the body parts on the screen. Although the participants were instructed to imagine that the body on the screen was a mirror image of themselves, this task could be carried out by means of activating memories of the body or also by referring to a prototypical representation of a human body. However, the cognitive process activated for this task is conscious, in contrast to the automatic, pre-reflexive processes involved in the BST. With regard to this last aspect, one may think that the BST also measures the Body Schema, namely “a system of postural and sensory–motor capacities that usually functions without perceptual monitoring” [98] (p. 26). However, as already discussed, any attempts to rigidly distinguish the various components of body representation have limitations [98], an issue which has also been reported in neurological patients with mixed symptoms [99].

The efficacy of motor training in restoring body representations has already been shown in a recent study carried out by the authors [47]. In the present paper, we confirm these data and demonstrate that motor imagery may also contribute to the recovery of body representations. However, our results show that the body representation of lower limbs had only been recovered at the follow-up assessment, suggesting the existence of a slow paced neuroplasticity that is in contrast with previous findings [47]. This suggests that various top-down and bottom-up mechanisms probably underlie the updating of body representations [100].

Taken as a whole, these data confirm that body representations in SCI can be somato-topically recovered by means of motor training, but also confirm that the mechanisms and times relating to this recovery remain for the most part unknown.

### 4.3. Pathological Below-Lesion Sensations and Better Clinical Scores Facilitate Body and Peripersonal Space Recovery in MI Training

Greater intensity and frequency of spasms seem to be connected with a better peripersonal space and body representation recovery after Motor + MI training.

Even if apparently counter-intuitive, this result is similar to previous data that indicate a potentially protective effect of pain (in particular neuropathic and visceral pain) in terms of maintaining self-body perception and reducing the presence of corporeal illusions and misperception [21]. In SCI individuals with complete lesions, spasms are the only painful sensations that can be felt from the below-lesional part of the body. Therefore, these also constitute the only feedback available to the individuals which enable them to maintain a sort of cognitive representation, albeit distorted, of their body. Spasms are reported in the below-lesional part of the body and we can hypothesise that these might work as surrogates of bodily sensations that in connection with MI training can improve Body and peripersonal space representations.

As expected, also lower and more recent lesions are connected with better or more stable recovery after MI training, as has already been demonstrated for corporeal illusions [21]. 

### 4.4. Limitations

One limitation of the study is the small sample size, which is, unfortunately, typical in rehabilitation studies. A further limitation is the variegate clinical condition of the participants, which limits the generalization of the findings. However, the impact of both these aspects was mitigated by the use of Bayesian Statistics, known to be adapt for small samples and resilient to extreme values [101].

## 5. Conclusions

Motor + MI training had positive effects on peripersonal space recovery in the SCI participants in this study. This improvement did not, however, last to the follow-up assessment suggesting the extremely plastic nature of peripersonal space representation.

Body representation was recovered only at the Follow-Up assessment, without any clear difference between those participants who were involved in the Motor + MI training and those who were only involved in the Motor Training. This indicates that motor training alone was sufficient and that the Body representation evaluated by means of the Body Sidedness Effect is not constantly updated.

Finally, there were clinical aspects which interacted with the Motor + MI training, suggesting that lower lesions (NLI) and more recent lesions have a better possibility of recovery, but that also more intense and frequent spasms led to similar positive outcomes.

These results shed new light on the role of Motor Imagery in rehabilitative training, in particular in cognitive representations such as Body and Peripersonal space representations. Moreover, they also raise interesting theoretical considerations concerning the nature of neuroplastic mechanisms in SCI individuals.

## Figures and Tables

**Table 1:** Clinical and demographic data of the participants in the study.

ID	Age (Years) ^a^	Lesion Onset (Years) ^b^	N. Treat. ^c^	NLI ^d^	AIS ^e^	Group ^f^	Motor ^g^	Gender ^h^
Subj01	43	26.82	10	T4	A	Motor	EKSO	M
Subj02	37	8.83	10	T4	A	Motor + MI	EKSO	M
Subj03	54	30.05	10	L1	D	Motor	EKSO	M
Subj04	65	29.05	10	T6	A	Motor + MI	EKSO	M
Subj05	44	18.24	8	T6	A	Motor	EKSO	M
Subj06	44	28.31	8	T7	A	Motor	EKSO	M
Subj07	65	29.35	10	T4	A	Motor + MI	EKSO	M
Subj08	57	1.63	10	C5	C	Motor	EKSO	M
Subj09	44	27.40	8	T4	A	Motor + MI	Mobilisation	M
Subj10	65	29.54	9	T6	A	Motor	Mobilisation	M
Subj11	54	30.58	9	L1	D	Motor + MI	Mobilisation	M
Subj12	39	5.58	9	T7	A	Motor	Mobilisation	M
Subj13	44	26.58	8	T6	A	Motor	Mobilisation	F
Subj14	49	15.64	10	T4	A	Motor + MI	Mobilisation	F
Subj15	65	10.64	10	T5	A	Motor + MI	Mobilisation	M
Mean	51.22	21.22	9.33	T = 12	A = 12	Motor = 8	EKSO = 8	M = 13
St. Dev.	9.88	9.81	0.94	L = 2	C = 1	Motor + MI = 7	Mobilisation = 7	F = 2
				C = 1	D = 2			

^a^ Age refers to the participants’ ages at the beginning of the training sessions; ^b^ Lesion Onset is the interval between the lesion onset to the beginning of the training sessions (expressed in years); ^c^ N. Treat.—the number of rehabilitation sessions (see Methods section for further details); ^d^ NLI–Neurological Level of Injury, that is the most caudal level of the spinal cord with totally spared somato-sensory functions [13]; ^e^ AIS is the ASIA Impairment Scale, A—Complete lesion; C-D—Incomplete lesions with sensory and some motor functions spared below the lesion [48]; ^f^ Group indicates whether the subject participated in the Motor or Motor + MI treatment (see Methods section for further details); ^g^ Motor indicates whether motor training of the participant was done by means of exoskeleton (EKSO), or by means of passive mobilisation (Mobilisation, see Methods section for further details); ^h^ M—males; F—females. The rows at the bottom of the table summarise the frequencies of the Thoracic, Lumbar and Cervical lesions, the number of subjects who participated in the group who did only motor treatment or in the group who did motor treatment and motor imagery, and the total number of males (M) and females (F).

**Table 2:** Results for the Bayesian model for the LLCCT evaluations divided into: (A) REAL condition and (B) VOID condition.

(A) REAL Condition	Mode ^a^	HDI ^b^		n_eff_^c^	Ȓ ^d^	BF_10_^e^	
(Intercept)	11.065	7.238	14.390	50	1.065	>150	H1 ^g^
Space	0.902	−2.510	4.101	221	1.016	0.409	
Training	−5.116	−8.177	−1.961	82	1.040	>150	H1
Time	11.022	6.572	16.483	208	1.014	>150	H1
Time^2 f^	−15.906	−22.293	−11.308	135	1.019	>150	H1
Space:Group	1.051	−2.027	5.046	36	1.088	0.422	
Space:Time	1.285	−3.052	6.456	171	1.017	0.543	
Space:Time^2 f^	3.766	−1.567	9.287	363	1.008	1.472	
Group:Time	−8.082	−12.878	−3.068	51	1.058	53.021	H1
Group:Time^2 f^	−6.824	−11.620	−0.643	140	1.038	7.306	H1
Space:Group:Time	−0.748	−6.254	3.970	92	1.036	0.642	
Space:Group:Time^2 f^	−12.339	−17.981	−6.478	92	1.034	>150	H1
**(B) VOID Condition**	**Mode**	**HDI**		**n_eff_**	**Ȓ**	**BF_10_**	
(Intercept)	15.081	11.298	18.621	49	1.075	>150	H1
Space	6.492	3.393	9.863	69	1.045	>150	H1
Group	21.305	18.552	25.146	44	1.073	>150	H1
Time	−0.797	−5.915	4.551	438	1.007	0.551	
Time^2 f^	3.886	−1.416	9.363	306	1.009	1.3	
Space:Group	0.030	−3.360	3.379	95	1.031	0.383	
Space:Time	−3.198	−8.014	1.354	307	1.011	1.341	
Space:Time^2 f^	−2.388	−7.872	2.251	36	1.086	0.911	
Group:Time	−5.268	−10.403	−0.286	252	1.019	4.224	
Group:Time^2 f^	−8.540	−14.874	−3.619	563	1.011	28.343	H1
Space:Group:Time	−9.152	−14.173	−4.731	109	1.031	>150	H1
Space:Group:Time^2 f^	6.894	2.015	11.763	208	1.014	16.964	H1

^a^ Mode refers to the mode of the posterior distribution; ^b^ HDI is the 95% Highest Density Interval [72] (pp. 87–89) of the posterior distribution; ^c^ n_eff_—Effective Number of Simulation draws; ^d^ Ȓ—Gelman-Rubin diagnostic index; ^e^ BF_10_—Bayes Factor, with the numerator representing the alternative hypothesis and the denominator representing the null hypothesis. The final column indicates whether the BF_10_ sustains the null (H0) or the alternative (H1) hypothesis; ^f^ Time^2^ is the quadratic effect of the three timepoints which was necessary to capture non-linear effects. Intercept is the intercept of the Generalised Multilevel Linear Models, Time is the linear effect of the three timepoints (T0, T1, T2); ^g^ H1—alternative hypothesis; H0 = null hypothesis.

**Table 3:** Effects of clinical variables in modulation of PPS around lower limbs. Description as in Table 2.

	Mode ^a^	HDI ^b^		n_eff_^c^	Ȓ ^d^	BF_10_^e^	
(Intercept)	78.867	69.795	87.956	73	1.045	>150	H1
Group	2.704	−7.591	10.886	190	1.016	1.11	
NLI ^f^	−0.684	−8.971	9.640	52	1.059	0.911	
Lesion Onset	0.349	−9.810	9.942	30	1.100	1.038	
PSFS–Frequency ^g^	−0.733	−10.907	7.794	67	1.045	1.013	
PSFS–Intensity ^h^	−1.163	−9.808	9.617	32	1.093	1.031	
VMIQ2 ^i^	−0.481	−11.213	9.385	151	1.022	1.035	
Group: NLI	25.995	16.680	34.995	23	1.152	>150	H1
Group: Lesion Onset	−3.457	−12.678	7.139	33	1.090	1.218	
Group: PSFS–Frequency	43.598	35.118	54.388	37	1.081	>150	H1
Group: PSFS–Intensity	10.226	−0.297	19.338	18	1.199	5.574	H1
Group: VMIQ2	−4.562	−14.945	5.441	17	1.204	1.552	

^a^ Mode refers to the mode of the posterior distribution; ^b^ HDI is the 95% Highest Density Interval [72] (pp. 87–89) of the posterior distribution; ^c^ n_eff_—Effective Number of Simulation draws; ^d^ Ȓ—Gelman-Rubin diagnostic index.; ^e^ BF_10_—Bayes Factor, with the numerator representing the alternative hypothesis and the denominator representing the null hypothesis. The final column indicates whether the BF_10_ sustains the null (H0) or the alternative (H1) hypothesis; ^f^ NLI—Neurological Level of Injury, that is the most caudal level of the spinal cord with totally spared somato-sensory functions [13]; ^g,h^ PSFS—Penn Spasms Frequency Scale [49]; ^i^ VMIQ2—Vividness of Motor Imagery Questionnaire 2—version modified in [29].

**Table 4:** Table of the results for the Bayesian model referring to the BST evaluations.

	Mode ^a^	HDI ^b^		n_eff_^c^	Ȓ ^d^	BF_10_^e^	
Intercept	3.103	1.046	4.636	9224	1.009	14.217	H1 ^g^
Background	−0.195	−1.970	1.584	123	1.022	0.179	H0 ^g^
Group	0.871	−1.154	2.671	111	1.026	0.285	
Time	0.489	−2.539	3.773	461	1.008	0.339	
Time^2 f^	−3.917	−6.656	−0.300	1498	1.003	3.509	
Background:Group	3.771	1.920	5.579	1118	1.002	>150	H1
Background:Time	−0.071	−3.136	2.918	254	1.012	0.301	
Background:Time^2 f^	−5.058	−8.205	−1.844	128	1.023	40.947	H1
Group:Time	−2.231	−5.506	0.727	89	1.035	0.939	
Group:Time^2 f^	3.846	0.795	7.472	484	1.009	6.759	H1
Background:Group:Time	−1.705	−4.785	1.386	186	1.014	0.554	
Background:Group:Time^2 f^	−0.807	−3.958	2.202	113	1.024	0.367	

^a^ Mode refers to the mode of the posterior distribution; ^b^ HDI is the 95% Highest Density Interval [72] (pp. 87–89) of the posterior distribution; ^c^ n_eff_—Effective Number of Simulation draws; ^d^ Ȓ—Gelman-Rubin diagnostic index.; ^e^ BF_10_—Bayes Factor, with the numerator representing the alternative hypothesis and the denominator representing the null hypothesis. The final column indicates whether the BF_10_ sustains the null (H0) or the alternative (H1) hypothesis; ^f^ Time^2^ is the quadratic effect of the three timepoints which was necessary to capture non-linear effects. Intercept is the intercept of the Generalised Multilevel Linear Models, Time is the linear effect of the three timepoints (T0, T1, T2); ^g^ H1—alternative hypothesis; H0 = null hypothesis.

**Table 5:** Results from the Bayesian model referring to the BSE evaluations with the FOOT background trials at T1, co-varying with NLI, PSFS and VMIQ-2. Description as in Table 2.

	Mode ^a^	HDI ^b^		n_eff_^c^	Ȓ ^d^	BF_10_^e^	
(Intercept)	58.370	50.798	66.666	227	1.015	>150	H1
Group	19.090	12.301	27.211	139	1.023	>150	H1
NLI ^f^	−16.028	−23.255	−7.589	474	1.007	>150	H1
Lesion Onset	−4.523	−12.051	3.437	38	1.079	1.455	
PSFS–Frequency ^g^	−10.990	−18.301	−3.227	82	1.034	31.577	H1
PSFS–Intensity ^h^	−0.584	−8.313	7.232	87	1.032	0.84	
VMIQ2 ^i^	−2.562	−10.511	4.970	70	1.040	1.009	
Group: NLI	1.654	−6.306	9.293	269	1.013	0.858	
Group: Lesion Onset	7.846	0.517	16.269	39	1.077	6.497	H1
Group: PSFS–Frequency	9.857	1.792	17.298	72	1.038	16.731	H1
Group: PSFS–Intensity	−16.872	−25.035	−9.497	236	1.011	>150	H1
Group: VMIQ2	−7.348	−15.040	0.324	93	1.031	4.232	

^a^ Mode refers to the mode of the posterior distribution; ^b^ HDI is the 95% Highest Density Interval [72] (pp. 87–89) of the posterior distribution; ^c^ n_eff_—Effective Number of Simulation draws; ^d^ Ȓ—Gelman-Rubin diagnostic index; ^e^ BF_10_—Bayes Factor, with the numerator representing the alternative hypothesis and the denominator representing the null hypothesis. The final column indicates whether the BF_10_ sustains the null (H0) or the alternative (H1) hypothesis; ^f^ NLI—Neurological Level of Injury, that is the most caudal level of the spinal cord with totally spared somato-sensory functions [13]; ^g,h^ PSFS—Penn Spasms Frequency Scale [49]; ^i^ VMIQ2–Vividness of Motor Imagery Questionnaire 2—modified version in [29].

**Figure A1:**
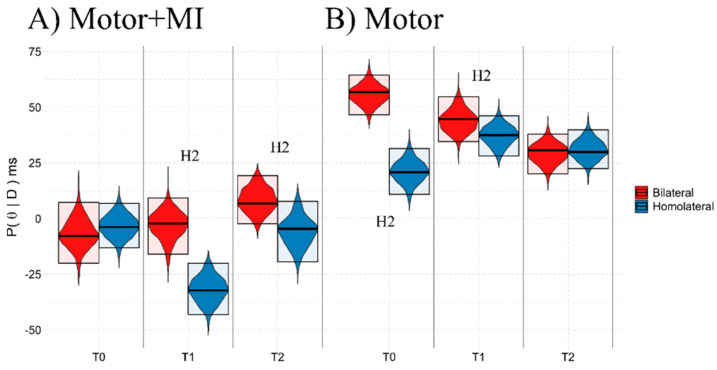
Graphical representation of the marginal posterior distributions of the Space:Condition:Time interaction in the VOID condition. The violin plots represent the marginal posterior distribution of the Bayesian model, the upper and lower boundaries of the box show the limits of the 95% HDI, while the central line represents the distribution mode. On the y−axis, the marginal posterior distribution (P(θ|D)), in milliseconds, represents the performance of the participants, and the x−axis shows the Time points: T0 (pre training), T1 (post training) and T2 (follow-up). (**A**) = Motor + MI, (**B**) = Motor. H1 = Pr(x > 0) > 83.5%, meaning that Homolateral > Bilateral, index of PPS representation. H2 = Pr(x > 0) < 16.5%, meaning that Bilateral > Homolateral, and with no PPS representation.

## Data Availability

All data and code are available at https://osf.io/5zjcv/, accessed on 8 September 2021.

## Appendix A

Outliers were removed for each participant and these were identified by means of the Interquartile Range Rule (considering the outliers to be the values outside the following range: first quartile—IQR * 1.5, third quartile + IQR * 1.5). Bayesian models were written in JAGS 4.3.0 [102] and interfaced in R 4.0.0 [103] by means of the jagsUI 1.5.1 package [104]. All models were fitted by means of four Markov Chains Monte Carlo (MCMC) [105]. These are sampling algorithms, that work by means of the autojags function that automatically increases the number of MCMC iterations until the Gelman and Rubin diagnostic index (Ȓ) [106] is less than 1.1 (Ȓ < 1.1). This guarantees that the MCMCs are stationary, namely, that the chains have reached a stable and reliable estimation of the parameters of the model. The autojags function starts with a minimum of 100 adaptive and 5000 normal MCMC iterations, to a maximum of 10,000 adaptative and 100,000 normal MCMC iterations, with steps of 100 adaptive and 5000 normal MCMC iterations. The burn-in iterations were fixed at 2000.

## Appendix B

The JAGS models used to analyse the VMIQ2, PSFS, MAS, MRC, NLI data (Ordinal model) and age and Lesion Onset (Robust Linear model).

In the JAGS code, the likelihood parts (lines 1–22) estimate the mean as the linear combination of the independent variables (mu). This value is then used to compute the probabilities of getting each ordinal level according to the thresholds. Then, these probabilities are used in the likelihood function and the categorial distribution.

Lines 27–30: the thresholds which are used to cut ??off the underlying normal distribution in the ordinal scores are estimated and ordered.

Lines 32–35: the estimates of the posterior distributions of the effects.

Lines 38–39: the computations of the scores to calculate the Posterior Predictive *p*-value.

In the JAGS code, the likelihood loop (lines 1–13) estimates the mean as the linear combination of the independent variables (mu) that will be used as the mean parameter of the student t distribution, with the variance sigma coming from a gamma distribution with alpha = 0.1 and beta = 0.1 (line 20) and degrees of freedom coming from another gamma distribution with the same characteristics (line 21).

Lines 16–18: the estimate of the posterior distributions of the effects.

Lines 24–25: the computation of the scores to calculate the Posterior Predictive *p*-value.

## Appendix C

JAGS model for the Generalised Multilevel Linear Model for Congruency Effects based on Reaction Times:

The underlying logic of this model naturally follows the computation of Congruency Effects.

Generally, a Congruency Effect is computed as reported in Equation (A1):

Congruency Effect = Average (Reaction Times of Incongruent Trials) − Average (Reaction Times of Congruent Trials), (A1)

It should be noted that this simple computation has some disadvantages:

1. computing the average of reaction times we are implicitly assuming that they are normally distributed, when they are not [83];
2. also when computing the differences between the averages, we do not consider the whole data set, with the consequence that these averages are more prone to outliers and the power of the analysis is thus weaker (1 − β).

To overcome these problems we can reasonably consider the two following simplified multilevel linear models (Equations (A2) and (A3)):

Reaction Times of Incongruent Trials ~ Population-level effects for Incongruent Trails ∗ X + Group-level effects for Incongruent Trials ∗ Z, (A2)

Reaction Times of Congruent Trials ~ Population-level effects for Congruent Trails ∗ X + Group-level effects for Congruent Trials ∗ Z, (A3)

where X is the contrast matrix of the independent variables for the Population-level effects, and Z the contrast matrix for the independent variables for the Group-level effects.

The Population- and Group-level effects of the Congruency Effect can be computed as follows (Equation (A4)):

Congruency Effects ~ (Population-level effects for Incongruent Trails-Population-level effects for Congruent Trails) ∗ X + Group-level effects ∗ Z. (A4)

This model has already been applied in [26].

In the JAGS code, the likelihood parts (lines 1–31) estimate the mean as the linear combination of the independent variables, which are used to estimate the mean of the Gaussian part of the ex-Gaussian distribution, with a variance of 100. The exponential part of the ex-normal distribution is exponentially distributed with lambda coming from a uniform prior distribution from 0.01 to 100 (line 39).

The Population-level effects of the independent factors come from Gaussian distributions with mean 0 and standard deviation 5 (variance 25, precision 1/25).

From line 41 to 62, there is the estimation of the Group-level effects. The inverse variance-covariance matrix of random effects comes from a Wishart distribution, while their means are from Gaussian distributions with mean 0 and variance 1000 (precision 1/1000).

From line 64 to line 69, there is the derivation of the indexes used to estimate the Posterior Predictive *p*-value.

For data and further details, please check the data availability statement.

Likelihood distributions:

Reaction Times ∼ Ex-Gaussian (μ, σ = 10, λ),
If Congruent trials:
μ = Xβ + Zξ with β being the population- and ξ the group-level effects,
If Incongruent trails:
μ = X(β_Congruency Effect_ + β_Congruent Trials_)+ Zξ (A5)

This is the likelihood distribution for Reaction Times for both Congruent and Incongruent trials.

Please, consider that if

μ_Congruency Effect_ = X(β_Incongruent Trial_ − β_Congruent Trial_) + Zξ, (A6)

Then:

μ_Incongruent_ = X(β_Congruency Effect_ + β_Congruent_) + Zξ, (A7)

Prior distributions:

β ~ Gaussian (0, 25),
λ ~ Uniform (0.01, 100),
ξ ~ MultiGaussian (ξ_μ_, Ω), (A8)

Hyperprior distributions:

Ω ~ Wishart^−1^ (diag(n), n) with n being the number of group-level effects,
ξ_μ_ ~ Gaussian(0, 1000), (A9)

## Appendix D

JAGS model for the Generalised Multilevel Linear Model for Congruency Effects based on Reaction Times without group-level effects:

This model differs from the model in Appendix B due to the fact that it does not have group-level effects.

## Appendix E

It is possible to observe that Bilateral trials were slower than Homolateral trials in the “Motor + MI” group at T1 (Pr(x > 0) = 0%, Bilateral = −2.19 [−16.03, 9.28], Homolateral = −32.23 [−43.06, −20.08]) and T2 (Pr(x > 0) = 5.62%, Bilateral = 6.73 [−2.34, 19.33], Homolateral = −4.57 [−19.41, 7.76]), while at T0 no clear effect was present (Pr(x > 0) = 67.22, Bilateral = −7.85 [−20.02, 7.26], Homolateral = −3.80 [−13.11, 6.88]).

Bilateral trials were slower than Homolateral trials also in the “Motor” group at T0 (Pr(x > 0) = 0%, Bilateral = 56.77 [46.62, 64.40], Homolateral = 20.86 [10.91, 31.49]) and T1 (Pr(x > 0) = 16.27%, Bilateral = 44.68 [34.59, 54.71], Homolateral = 37.47 [28.16, 46.15]), while at T2 no clear outcome was observable (Pr(x > 0) = 59.39, Bilateral = 30.75 [20.16, 37.99], Homolateral = 29.93 [22.42, 39.93]).

For a graphical representation see Figure A1.
